# Cytoarchitectural changes in the olfactory bulb of Parkinson’s disease patients

**DOI:** 10.1038/npjparkd.2016.11

**Published:** 2016-06-09

**Authors:** John W Cave, Nana Fujiwara, Ava R Weibman, Harriet Baker

**Affiliations:** 1 Burke Medical Research Institute, 785 Mamaroneck Avenue, White Plains, NY, USA; 2 The Feil Family Brain and Mind Research Institute, Weill Cornell Medical College, 1300 York Avenue, New York, NY, USA

## Abstract

Olfactory dysfunction is associated with nearly all the cases of Parkinson’s disease (PD) and typically manifests years before motor symptoms are detected. The cellular mechanisms underlying this dysfunction, however, are not understood. In this study, olfactory bulbs (OBs) from male control and PD subjects were examined by histology for changes in cytoarchitecture. These studies found that the general OB laminar organization and the number of interneurons expressing tyrosine hydroxylase were unaltered. In contrast, the number of mitral/tufted projection neurons and interneurons expressing Calretinin were significantly decreased in PD subjects. This study reveals changes in OB cytoarchitecture mediated by PD and provides valuable insight into identifying specific OB neuronal populations vulnerable to PD-related neurodegeneration.

## Introduction

**Figure 1:**
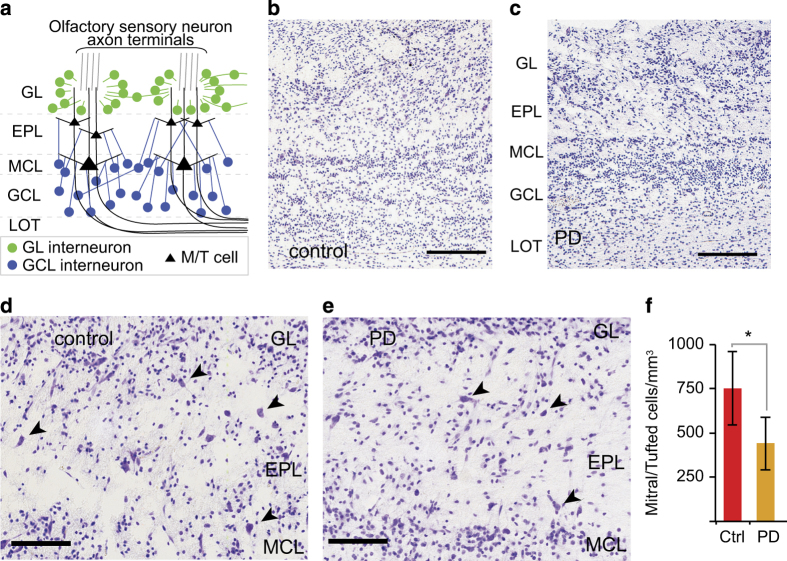
The number of M/T cells is decreased in PD patients even though OB laminar organization is not disrupted. (**a**) OB laminar organization. (**b**,**c**) Cresyl-violet-stained sections show that the general OB laminar organization is similar between control and PD patients. Bar = 250 μm. (**d**,**e**) Higher magnification images of cresyl-violet-stained sections in control and PD patients, respectively. Selected M/T cells are highlighted by arrowheads. Bar = 100 μm. (**f**) M/T cell densities were significantly decreased in PD subjects (asterisk indicates *P*<0.05). EPL, external plexiform layer; GCL, granule cell layer; GL, glomerular layer; LOT, lateral olfactory tract; MCL, mitral cell layer; M/T, mitral and tufted; OB, olfactory bulb; PD, Parkinson’s disease.

Odorant sensory information generated by olfactory sensory neurons (OSN) is first processed in the main olfactory bulb (OB). Odorant information is relayed from OSN axons to mitral and tufted (M/T) cells, and then transmitted by M/T cells to other cortical regions. The input to, and output from, M/T cells are modulated by OB inhibitory interneurons. Altogether, the M/T cells and interneurons are organized in distinct lamina (Figure 1a).

The OB is one of the earliest regions to show accumulation of Lewy bodies containing α-synuclein.^1^ Consistent with a pivotal role in the onset of PD, nearly all (~90%) cases of sporadic PD are associated with olfactory dysfunction that occurs several years before motor symptoms are noticeable.^2^ The cellular basis for PD-associated olfactory dysfunction, however, has not been elucidated. In this study, we have examined OB tissue from control and PD subjects by histological methods to establish whether OB cytoarchitecture is disrupted by PD.

## Results

Cresyl-violet-stained sections revealed no substantial change in the general OB laminar organization between control and PD subjects (Figure 1b,c). To address whether specific cell types were affected, the number of M/T cells was counted in cresyl-violet-stained sections. Cresyl-violet-stained sections were used for this analysis because immunohistochemical staining with M/T-specific markers Tbx21 and Reelin was inconsistent in quality, possibly owing to variations in post-mortem intervals (Supplementary Information). This analysis showed that the OB of PD subjects had significantly fewer M/T cells when compared with controls (Figure 1d–f).

**Figure 2:**
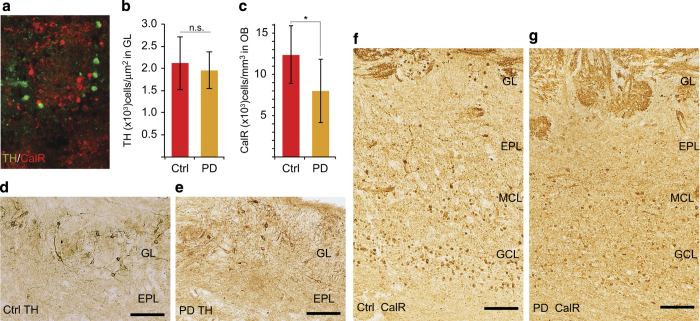
PD differentially affects OB interneuron populations. (**a**) Immunofluorescence staining shows that TH and CalR are expressed by mutually exclusive OB interneuron subpopulations. (**b**,**c**) The density of TH-expressing cells in the glomerular layer was not significantly different (NS), but PD subjects had significantly lower density of CalR-expressing interneurons in the OB (asterisk indicates *P*<0.05). (**d**–**g**) Immunohistochemistry of TH and CalR expression, respectively, in control and PD subjects. Bar = 100 μm. CalR, calretnin; NS, not significant; OB, olfactory bulb; PD, Parkinson’s disease; TH, tyrosine hydroxylase.

To address whether specific OB interneuron subpopulations were also altered in PD subjects, immunohistochemistry was used to examine tyrosine hydroxylase (TH) and calretnin (CalR) expression. These proteins are expressed by mutually exclusive OB interneuron subpopulations (Figure 2a). TH-expressing interneurons are limited to the glomerular layer (Figure 2d,e), whereas CalR is expressed in glomerular and granule cell layer interneurons as well as olfactory sensory axons (Figure 2f,g). Cell counts revealed that PD subjects had no significant change in the number of TH-expressing glomerular layer cells, but there was a significant reduction in the number of CalR-containing cells in the combined glomerular and granule cell layers.

## Discussion

The PD-related decrease in M/T and CalR-containing cells observed in these studies is the first to show the loss of specific OB cell types in the OB of PD subjects. The loss of these cell types would be expected to adversely affect both odorant detection threshold and discrimination, but they do not rule out the possibility that PD-mediated neurodegeneration also occurs in other cortical regions associated with processing odorant sensory information.

The findings of this study are consistent with, and complement, a previous report with human OB tissue that showed Lewy bodies containing α-synuclein preferentially accumulate in both mitral cells and cells expressing calcium-binding proteins, such as CalR, but not in TH-positive cells.^3^ Our observation that there is no significant change in the number of TH-expressing OB interneurons in male PD patients is also consistent with previous analyses of only male subjects.^4^ Previous studies that incorporated female subjects, however, have reported significant increases in the number of TH-expressing OB interneurons in PD patients.^4–6^ Thus, PD appears to have differential gender-based effects on the number of TH-expressing neurons in the OB. The data are consistent, however, in that PD does not diminish the number of TH-expressing cells in the OB.

The decrease in M/T and CalR-containing cells suggests that these cell types are vulnerable to PD-mediated neurodegeneration. It is possible, however, that only one of these cell types is directly vulnerable and its reduction results in the loss of the other. Relatedly, the present study cannot distinguish whether M/T and CalR cells are lost concomitantly or whether they are lost at different times during the progression of the disease. Additional limitations to the current study include the relatively small sample size and the lack of patient-specific medical histories (such as disease stage, olfactory testing, genetic background, smoking, and medication histories). Future histological studies with OB tissue from control and PD subjects that address these limitations will further improve our understanding of PD-mediated changes in OB cytoarchitecture. Establishing the OB neuronal populations vulnerable to PD-mediated degeneration will advance our understanding of what makes specific cell types susceptible to this disease. Molecular comparisons between vulnerable OB and midbrain dopaminergic neurons may reveal critical features shared by these neurons and allow for development of novel biomarkers to facilitate early-stage detection of PD.

## Materials and methods

Detailed methods are available in the online Supplementary Information.
