# Supplementary material for: Cytoarchitectural changes in the olfactory bulb of Parkinson’s disease patients
Source: NPJ Parkinsons Dis. 2016 Jun 9;2:16011–. doi: 10.1038/npjparkd.2016.11 (PMC5516575; doi:10.1038/npjparkd.2016.11)
Supplement: Supplementary Information [file npjparkd201611-s1.doc]

**Supplementary Information**

***Methods***

*Olfactory Bulb Tissue*

Human olfactory bulb (OB) tissue was collected post-mortem by the Harvard Brain Tissue Resource Center. All patient data and informed consent was collected and maintained by the Harvard Brain Tissue Resource Center. Work with human tissue at the Burke Medical Research Institute was approved by the Burke Rehabilitation Hospital Committee for Human Rights in Research (protocol BRC-473). All subjects examined in this study were male. Control OB tissue was from subjects with no previous diagnosis of neurodegenerative disease and did not show signs of neurodegenerative disease during autopsy. All Parkinson’s disease (PD) tissue was from subjects with a clinical diagnosis prior to death that was confirmed at autopsy. Both control and PD tissues were supplied with unique subject identification numbers, but no other patient information was provided. Sample size was limited by the amount of tissue available, and the amount provided for this study was sufficient to detect changes for values with variances up to 30%, alpha values of 0.1 or less, and power values of 70% or better. A summary of tissue provided for this study is shown in Table 1. Tissue was provided as formalin-fixed and was cut as 30 μm thick sagittal sections with a Leica CM 3050S cyrostat.

| **Subject ID** | **Group** | **Sex** | **Age** | **Post Mortem Interval (hours)** |
| --- | --- | --- | --- | --- |
| 8162 | control | M | 64 | 17.8 |
| 8095 | control | M | 70 | 22.9 |
| 7969 | control | M | 82 | 18.1 |
| 7959 | control | M | 78 | 21.9 |
| 8224 | PD | M | 83 | 13 |
| 8136 | PD | M | 76 | 12.3 |
| 8078 | PD | M | 66 | 22.5 |
| 7905 | PD | M | 66 | 21.4 |

**Table 1**. Donor subject information for the tissue examined in this study.

*Histology and Immunohistochemistry*

For cresyl violet staining, sections were additionally fixed in 4% paraformaldehyde for 20 minutes, then rinsed with water. Sections were sequentially dehydrated with 50%, 75%, 95% and 100% ethyl alcohol for 2, 3, 4 and 5 minutes, respectively. Sections were then immersed in a 1:1 solution of ethyl alcohol: chloroform for 10 minutes before being sequentially rehydrated with 100%, 95%, 75% and 50% alcohol solutions for 4, 3, 2, and 2 minutes, respectively. Following rehydration, sections were rinsed with water and then immersed in 0.125% cresyl violet solution for 4 minutes before being rinsed with water and sequentially dehydrated with 50%, 75%, and 95% ethyl alcohol for 1, 2, and 2 minutes, respectively. Sections were resolved with 95% ethyl alcohol with acetic acid for 1 minute before being placed in 100% ethyl alcohol twice for 2 minutes each time. Sections were then immersed twice in xylene for 5 minutes each time before being coated with Permount (Fisher Chemical) and cover-slipped.

All immunohistochemistry was visualized with diaminobenzadine staining using the Vectastain Elite ABC kit (Vector Laboratories). Sections were rehydrated in phosphate buffered saline (PBS) for 5 minutes then treated with 1% bovine serum albumin (BSA) in PBS for 1 hour. The 1% BSA solution was replaced with a 0.5% BSA solution in PBS that contained the primary antibody. Primary antibodies for TH and CalR were used at dilutions of 1:5000 (mouse; ImmunoStar 22941) and 1:2500 (goat; EMD Millipore AB1550), respectively. Sections were incubated with the primary antibodies overnight, then washed three times with a 0.5% BSA/PBS solution for 2 minutes each time. Sections were incubated for 1 hour with a 0.5% BSA/PBS solution containing a biotinylated antibody (at a 1:200 dilution; Vectastain Laboratories) raised against either mouse or goat antibodies. After the hour, sections were washed three times with a 0.5% BSA/PBS solution for 2 minutes each time. Sections were then treated with a PBS solution containing 0.3% hydrogen peroxide for 20 minutes before being were washed again three times with a 0.5% BSA/PBS solution for 2 minutes each time. Sections were incubated for 1 hour with a phosphate-buffered solution with streptavidin/biotin-conjugated horseradish peroxide (made with the Vectastain Elite ABC kit from Vector Laboratories following the manufacturer’s protocol). Sections were then washed three times with a 0.5% BSA/PBS solution for 2 minutes each time. The staining reaction was initiated with the application of a 1mg/mL solution of diaminobenzadine in phosphate buffer with 0.01% hydrogen peroxide. At the completion of the staining reactions, the sections were washed three times with a 0.5% BSA/PBS solution for 2 minutes each time. Sections were dehydrated with 50%, 75%, 95% and twice with 100% ethyl alcohol for 2, 3, 4 and 5 minutes, respectively, then immersed twice in xylene for 5 minutes each time before being coated with Permount (Fisher Chemical) and cover-slipped.

*Cell counting and Analysis*

Four control and PD subjects were used for analysis of either M/T, TH- or CalR-expressing cells. For each subject, a minimum of three sections with a minimum of 90μm spacing from the medial region of the OB were used. All sections were examined on a Zeiss Axiovert 200M microscope. All cell counts were made along the ventral side of the bulb. Target cells were counted in areas defined with StereoInvestigator software (MBF Bioscience). Target cell densities for each section were calculated by dividing the cell counts by the volume of the counting region. TH-expressing cells were counted in the glomerular layer, whereas CalR cell counts are a combination of cells found in both the glomerular and granule cell layers. For TH and CalR, all cells labeled by immunohistochemistry within the defined region were counted. For M/T cells, counting areas were confined to the external plexiform layer and superficial granule cell layers. For counting purposes, M/T cells were required to have cresyl violet-stained nuclei with minimum minor and major elliptical diameters of 10μm and 12μm, respectively. Individuals counting cells in the sections were blind to which patient group the sections being analyzed belong, but patient groups were known during the analyses of the cell count data. None of the counted sections were excluded the analysis. All error bars shown represent the standard deviation. The mean, the variance, and standard deviation for each the target cell density were calculated using Excel (Microsoft)(Tables 2-7). For all data comparisons shown in the figures, tests of statistical significance between control and PD groups were conducted with two-tailed Mann-Whitney tests using Prism (GraphPad) with significance threshold of *p* ≤ 0.05. Mann-Whitney tests were used for all analysis of target cell densities because the comparison of target cell densities between control and PD groups contain a dependent continuous variable, an independent variable between two groups, the cell counts for each patient group were independent, and distributions of cell densities for each group have similar distributions.

| **ID** | **Group** | **Section** | **Counting Area (µm^2^)** | **Section**  **Depth (µm)** | **Couting Volume (mm^3^)** | **Counted Cells** | **Cell Density**  **(cells/mm^3^)** |
| --- | --- | --- | --- | --- | --- | --- | --- |
| 7905 | control | 1 | 1112452 | 30 | 0.0334 | 20 | 599 |
|  |  | 2 | 1036042 | 30 | 0.0311 | 26 | 837 |
|  |  | 3 | 1099602 | 30 | 0.0330 | 16 | 485 |
|  |  | 4 | 1097088 | 30 | 0.0329 | 23 | 699 |
|  |  | 5 | 1012491 | 30 | 0.0304 | 26 | 856 |
|  |  | 6 | 998813 | 30 | 0.0300 | 22 | 734 |
|  |  | 7 | 859572 | 30 | 0.0258 | 19 | 737 |
| 8224 | control | 1 | 738849 | 30 | 0.0222 | 18 | 812 |
|  |  | 2 | 852665 | 30 | 0.0256 | 24 | 938 |
|  |  | 3 | 895781 | 30 | 0.0269 | 22 | 819 |
|  |  | 4 | 859339 | 30 | 0.0258 | 22 | 853 |
|  |  | 5 | 679412 | 30 | 0.0204 | 14 | 687 |
|  |  | 6 | 926520 | 30 | 0.0278 | 23 | 827 |
|  |  | 7 | 914605 | 30 | 0.0274 | 18 | 656 |
|  |  | 8 | 770970 | 30 | 0.0231 | 16 | 692 |
| 8136 | control | 1 | 717264 | 30 | 0.0215 | 17 | 790 |
|  |  | 2 | 724537 | 30 | 0.0217 | 18 | 828 |
|  |  | 3 | 693011 | 30 | 0.0208 | 28 | 1347 |
|  |  | 4 | 626814 | 30 | 0.0188 | 18 | 957 |
|  |  | 5 | 1034880 | 30 | 0.0310 | 26 | 837 |
|  |  | 6 | 845730 | 30 | 0.0254 | 17 | 670 |
|  |  | 7 | 724754 | 30 | 0.0217 | 10 | 460 |
|  |  | 8 | 677908 | 30 | 0.0203 | 16 | 787 |
| 8078 | control | 1 | 942599 | 30 | 0.0283 | 17 | 601 |
|  |  | 2 | 967491 | 30 | 0.0290 | 14 | 482 |
|  |  | 3 | 777995 | 30 | 0.0233 | 31 | 1328 |
|  |  | 4 | 1051740 | 30 | 0.0316 | 16 | 507 |
|  |  | 5 | 817615 | 30 | 0.0245 | 14 | 571 |
|  |  | 6 | 902666 | 30 | 0.0271 | 17 | 628 |
|  |  | 7 | 852126 | 30 | 0.0256 | 15 | 587 |
|  |  |  |  |  |  |  |  |
| 8162 | PD | 1 | 703178 | 30 | 0.0211 | 10 | 474 |
|  |  | 2 | 738701 | 30 | 0.0222 | 11 | 496 |
|  |  | 3 | 684585 | 30 | 0.0205 | 5 | 243 |
|  |  | 4 | 689482 | 30 | 0.0207 | 5 | 242 |
|  |  | 5 | 685647 | 30 | 0.0206 | 5 | 243 |
|  |  | 6 | 722293 | 31 | 0.0224 | 14 | 625 |
|  |  | 7 | 588788 | 30 | 0.0177 | 8 | 453 |
|  |  | 8 | 757243 | 30 | 0.0227 | 7 | 308 |
| 7959 | PD | 1 | 919590 | 30 | 0.0276 | 15 | 544 |
|  |  | 2 | 785380 | 30 | 0.0236 | 8 | 340 |
|  |  | 3 | 756259 | 30 | 0.0227 | 10 | 441 |
|  |  | 4 | 744660 | 30 | 0.0223 | 13 | 582 |
|  |  | 5 | 749546 | 30 | 0.0225 | 16 | 712 |
|  |  | 6 | 891803 | 30 | 0.0268 | 12 | 449 |
|  |  | 7 | 943569 | 30 | 0.0283 | 12 | 424 |
|  |  | 8 | 804690 | 30 | 0.0241 | 14 | 580 |
| 7969 | PD | 1 | 804366 | 30 | 0.0241 | 9 | 373 |
|  |  | 2 | 660526 | 30 | 0.0198 | 6 | 303 |
|  |  | 3 | 694800 | 30 | 0.0208 | 7 | 336 |
|  |  | 4 | 727386 | 30 | 0.0218 | 9 | 412 |
|  |  | 5 | 691152 | 30 | 0.0207 | 9 | 434 |
|  |  | 6 | 635254 | 30 | 0.0191 | 10 | 535 |
|  |  | 7 | 658918 | 30 | 0.0198 | 13 | 658 |
|  |  | 8 | 789653 | 30 | 0.0237 | 12 | 507 |
| 8095 | PD | 1 | 678481 | 30 | 0.0204 | 17 | 835 |
|  |  | 2 | 805008 | 30 | 0.0242 | 12 | 497 |
|  |  | 3 | 810094 | 30 | 0.0243 | 11 | 453 |
|  |  | 4 | 881863 | 30 | 0.0265 | 8 | 302 |
|  |  | 5 | 954374 | 30 | 0.0286 | 8 | 279 |
|  |  | 6 | 935973 | 30 | 0.0281 | 5 | 178 |
|  |  | 7 | 908303 | 30 | 0.0272 | 12 | 440 |

**Table 2**. Cell counting data for M/T cells.

| **Property** | **Control Subjects** | **PD**  **Subjects** |
| --- | --- | --- |
| Mean Cell Density (cells/mm^3^) | 754 | 442 |
| Variance | 43390 | 22478 |
| Standard Deviation | 208 | 150 |

**Table 3**. Mean, variance and standard deviation for M/T cell densities.

| **ID** | **Group** | **Section** | **Counting Area (µm^2^)** | **Section**  **Depth (µm)** | **Couting Volume (mm^3^)** | **Counted Cells** | **Cell Density**  **(cells/mm^3^)** |
| --- | --- | --- | --- | --- | --- | --- | --- |
| 8078 | control | 1 | 1571050 | 30 | 0.0471 | 119 | 2525 |
|  |  | 2 | 1861190 | 30 | 0.0558 | 134 | 2400 |
|  |  | 3 | 3538220 | 30 | 0.1061 | 165 | 1554 |
|  |  | 4 | 3634050 | 30 | 0.1090 | 227 | 2082 |
| 7905 | control | 1 | 2198071 | 30 | 0.0659 | 178 | 2699 |
|  |  | 2 | 462534 | 30 | 0.0139 | 39 | 2811 |
|  |  | 3 | 1822315 | 30 | 0.0547 | 115 | 2104 |
|  |  | 4 | 4839060 | 30 | 0.1452 | 236 | 1626 |
| 8224 | control | 1 | 3157058 | 30 | 0.0947 | 248 | 2618 |
|  |  | 2 | 2420821 | 30 | 0.0726 | 210 | 2892 |
|  |  | 3 | 1418070 | 30 | 0.0425 | 117 | 2750 |
|  |  | 4 | 2637488 | 30 | 0.0791 | 187 | 2363 |
| 8136 | control | 1 | 5990140 | 30 | 0.1797 | 204 | 1135 |
|  |  | 2 | 7344360 | 30 | 0.2203 | 250 | 1135 |
|  |  | 3 | 3631930 | 30 | 0.1090 | 184 | 1689 |
|  |  | 4 | 2783376 | 30 | 0.0835 | 129 | 1545 |
|  |  |  |  |  |  |  |  |
| 8162 | PD | 1 | 1597215 | 30 | 0.0479 | 114 | 2379 |
|  |  | 2 | 2329380 | 30 | 0.0699 | 160 | 2290 |
|  |  | 3 | 957605 | 30 | 0.0287 | 54 | 1880 |
|  |  | 4 | 2032601 | 30 | 0.0610 | 111 | 1820 |
| 8095 | PD | 1 | 1482596 | 30 | 0.0445 | 97 | 2181 |
|  |  | 2 | 2633100 | 30 | 0.0790 | 170 | 2152 |
|  |  | 3 | 1668909 | 30 | 0.0501 | 137 | 2736 |
|  |  | 4 | 3302480 | 30 | 0.0991 | 188 | 1898 |
| 7969 | PD | 1 | 5945791 | 30 | 0.1784 | 350 | 1962 |
|  |  | 2 | 6978250 | 30 | 0.2093 | 425 | 2030 |
|  |  | 3 | 4131741 | 30 | 0.1240 | 231 | 1864 |
|  |  | 4 | 2638560 | 30 | 0.0792 | 167 | 2110 |
| 7959 | PD | 1 | 7388200 | 30 | 0.2216 | 301 | 1358 |
|  |  | 2 | 8948540 | 30 | 0.2685 | 238 | 887 |
|  |  | 3 | 2689420 | 30 | 0.0807 | 156 | 1934 |
|  |  | 4 | 2241060 | 30 | 0.0672 | 128 | 1904 |

**Table 4**. Cell counting data for TH-expressing cells.

| **Property** | **Control Subjects** | **PD**  **Subjects** |
| --- | --- | --- |
| Mean Cell Density (cells/mm^3^) | 2120 | 1961 |
| Variance | 358561 | 170509 |
| Standard Deviation | 599 | 413 |

**Table 5**. Mean, variance, and standard deviation for TH-expressing cell densities.

| **ID** | **Group** | **Section** | **Counting Area (µm^2^)** | **Section**  **Depth (µm)** | **Couting Volume (mm^3^)** | **Counted Cells** | **Cell Density**  **(cells/mm^3^)** |
| --- | --- | --- | --- | --- | --- | --- | --- |
| 8078 | control | 1 | 3264920 | 30 | 0.0979 | 1366 | 13946 |
|  |  | 2 | 2658286 | 30 | 0.0797 | 1048 | 13141 |
|  |  | 3 | 2521209 | 30 | 0.0756 | 1050 | 13882 |
|  |  | 4 | 3459330 | 30 | 0.1038 | 1316 | 12681 |
| 7905 | control | 1 | 2729570 | 30 | 0.0819 | 1287 | 15717 |
|  |  | 2 | 3994270 | 30 | 0.1198 | 1657 | 13828 |
|  |  | 3 | 2587935 | 30 | 0.0776 | 1009 | 12996 |
|  |  | 4 | 5270900 | 30 | 0.1581 | 2075 | 13122 |
|  |  | 5 | 6375720 | 30 | 0.1913 | 2553 | 13348 |
| 8224 | control | 1 | 3766174 | 30 | 0.1130 | 2012 | 17808 |
|  |  | 2 | 4235478 | 30 | 0.1271 | 1120 | 8814 |
|  |  | 3 | 4352670 | 30 | 0.1306 | 1796 | 13754 |
|  |  | 4 | 2832780 | 30 | 0.0850 | 1306 | 15368 |
| 8136 | control | 1 | 5321770 | 30 | 0.1597 | 963 | 6032 |
|  |  | 2 | 5359200 | 30 | 0.1608 | 963 | 5990 |
|  |  | 3 | 4387230 | 30 | 0.1316 | 931 | 7074 |
|  |  |  |  |  |  |  |  |
| 8162 | PD | 1 | 2423015 | 30 | 0.0727 | 891 | 12257 |
|  |  | 2 | 1087980 | 30 | 0.0326 | 432 | 13236 |
|  |  | 3 | 2183830 | 30 | 0.0655 | 717 | 10944 |
|  |  | 4 | 2105210 | 30 | 0.0632 | 572 | 9057 |
| 8095 | PD | 1 | 2755020 | 30 | 0.0827 | 386 | 4670 |
|  |  | 2 | 4621950 | 30 | 0.1387 | 763 | 5503 |
|  |  | 3 | 3733990 | 30 | 0.1120 | 455 | 4062 |
|  |  | 4 | 2857510 | 30 | 0.0857 | 399 | 4654 |
| 7969 | PD | 1 | 12047900 | 30 | 0.3614 | 4722 | 13065 |
|  |  | 2 | 10723360 | 30 | 0.3217 | 3659 | 11374 |
|  |  | 3 | 11114460 | 30 | 0.3334 | 3520 | 10557 |
| 7959 | PD | 1 | 7687060 | 30 | 0.2306 | 1278 | 5542 |
|  |  | 2 | 9323630 | 30 | 0.2797 | 1211 | 4330 |
|  |  | 3 | 5802570 | 30 | 0.1741 | 460 | 2643 |

**Table 6**. Cell counting data for CalR-expressing cells.

| **Property** | **Control Subjects** | **PD**  **Subjects** |
| --- | --- | --- |
| Mean Cell Density (cells/mm^3^) | 12344 | 7992 |
| Variance | 12182474 | 14702676 |
| Standard Deviation | 3490 | 3834 |

**Table 7**. Mean, variance, and standard deviation for CalR-expressing cell densities.
